# Relative risk estimation of dengue disease at small spatial scale

**DOI:** 10.1186/s12942-017-0104-x

**Published:** 2017-08-15

**Authors:** Daniel Adyro Martínez-Bello, Antonio López-Quílez, Alexander Torres Prieto

**Affiliations:** 1 Departament d’Estadística i Investigació Operativa, Facultat de Matemátiques, Universitat de València, C/Dr Moliner 50, 46100 Burjassot, Valencia Spain; 2 Epidemiological Surveillance, Health Office of Department of Santander, Cl. 45 11-52, Bucaramanga, 680001 Colombia

**Keywords:** Disease mapping, Satellite images, Bayesian modeling, Cohen’s Kappa

## Abstract

**Background:**

Dengue is a high incidence arboviral disease in tropical countries around the world. Colombia is an endemic country due to the favourable environmental conditions for vector survival and spread. Dengue surveillance in Colombia is based in passive notification of cases, supporting monitoring, prediction, risk factor identification and intervention measures. Even though the surveillance network works adequately, disease mapping techniques currently developed and employed for many health problems are not widely applied. We select the Colombian city of Bucaramanga to apply Bayesian areal disease mapping models, testing the challenges and difficulties of the approach.

**Methods:**

We estimated the relative risk of dengue disease by census section (a geographical unit composed approximately by 1–20 city blocks) for the period January 2008 to December 2015. We included the covariates normalized difference vegetation index (NDVI) and land surface temperature (LST), obtained by satellite images. We fitted Bayesian areal models at the complete period and annual aggregation time scales for 2008–2015, with fixed and space-varying coefficients for the covariates, using Markov Chain Monte Carlo simulations. In addition, we used Cohen’s Kappa agreement measures to compare the risk from year to year, and from every year to the complete period aggregation.

**Results:**

We found the NDVI providing more information than LST for estimating relative risk of dengue, although their effects were small. NDVI was directly associated to high relative risk of dengue. Risk maps of dengue were produced from the estimates obtained by the modeling process. The year to year risk agreement by census section was sligth to fair.

**Conclusion:**

The study provides an example of implementation of relative risk estimation using Bayesian models for disease mapping at small spatial scale with covariates. We relate satellite data to dengue disease, using an areal data approach, which is not commonly found in the literature. The main difficulty of the study was to find quality data for generating expected values as input for the models. We remark the importance of creating population registry at small spatial scale, which is not only relevant for the risk estimation of dengue but also important to the surveillance of all notifiable diseases.

## Background

Dengue is an arboviral disease characterized by fever and vascular complications and is endemic in many tropical and subtropical regions of the world [1]. Within the global efforts to control the disease, representation of the problem in space and time is key to supporting disease surveillance systems [2]. Spatial and spatio-temporal models are useful for generating risk maps for dengue disease, supporting early warning systems and intervention programs [2, 3].

Disease mapping tools correlated dengue incidence with socioeconomic, demographic and environmental variables using the Moran index in Brazil [4], while its geographical distribution has been characterized using spatial statistics and geographical information system (GIS) analysis in Ecuador [5]. Dengue disease mapping has also been combined with surveillance and monitoring of the *Aedes aegypti* vector in the Middle East [6], and in Peru, investigators have explored the association between dengue and clinical, meteorological, climatic, and sociopolitical variables through fuzzy association rule mining in a spatial setting [7]. At a micro-regional scale, Lowe et al. [8] included temperature, rainfall, the El Niño Southern Oscillation index, and other relevant socioeconomic and environmental variables in a spatio-temporal Bayesian hierarchical model implemented with Markov Chain Monte Carlo (MCMC), generating predictions at spatial and temporal levels and supporting a dengue alert system. Honorato et al. [9] studied the relationship between risk of dengue and sociodemographic variables using Bayesian spatial regression models in the municipalities of Espírito Santo, Brazil, while Ferreira and Smith [10] modeled the number of cases of dengue fever in Rio de Janeiro, Brazil, considering the cases as a Poisson random variable, with conditional autoregressive (CAR) priors in the spatial random effects, testing different neighborhood structures and covariates with fixed coefficients.

Colombia is highly endemic for dengue disease. From 2000 to 2011, the country experienced a stable annual incidence of dengue, with major outbreaks in 2001–2003 and 2010, followed by a considerable decrease of incidence in 2011, with cases mainly occurring in children (${<}15$ years of age) and the highest incidence in 2009 in infants (${<}1$ year of age) [11]. Small scale studies using dengue reports have investigated the spatial autocorrelation of dengue cases [12] and the association between dengue and satellite environmental data using spatially stratified tests of ecological niche models [13]. Hagenlocher et al. [14] performed a spatial assessment of current socioeconomic vulnerabilities to dengue fever in 340 neighborhoods of a Colombian city through a spatial approach that included expert-based and purely statistical-based modeling of current vulnerability levels using a GIS.

At national level, Quintero et al. [15] used epidemiological surveillance data (weekly cases) and Poisson regression models to assess the influence of the El Niño Southern Oscillation index and pluviometry on dengue incidence, adjusting by year and week.At a regional scale, Cadavid-Restrepo et al. [16] explored the variation in spatial distribution of notified dengue cases in Colombia from 2007 to 2010, exploring associations between the disease and selected environmental risk factors through a Bayesian spatio-temporal conditional autoregressive model. The results elucidate the role of environmental risk factors in the spatial distribution of dengue disease, explaining how these factors can be used to develop and refine preventive approaches for dengue in Colombia. All these studies are strategic to the research of surveillance and control of dengue disease, demonstrating the importance of representation of the disease in space and time.

In Colombia, dengue epidemiological surveillance is based in passive notification of cases, coordinated by the ‘Instituto Nacional de Salud’ (Colombia National Institute of Health) [17], and supports monitoring, prediction, risk factor identification and intervention measures. Even though the surveillance network works adequately, and provides information to all the national institutions involved in dengue control, we appreciate that disease mapping techniques currently developed and employed for many health problems are not widely applied.

We selected the Colombian city of Bucaramanga to use Bayesian areal disease mapping models, testing the challenges and difficulties of the approach to dengue disease mapping. Bucaramanga was one of the cities with the highest dengue incidence in Colombia by year during the period 2008–2015. We estimated the relative risk of dengue disease, applying Bayesian spatial areal models to dengue case counts and satellite covariates (normalized difference vegetation index (NDVI) and land surface temperature (LST)) with fixed and space-varying coefficients, at a small spatial scale and with global and annual aggregation time scales, for the 2008–2015 period. Ideally, we would use data on the vector presence, distribution and ecology, but that kind of data does not exist for the city of Bucaramanga at the aggregation level of the study. We relied on satellite images to search associations between dengue incidence and environmental data. In addition, we provided a Bayesian model to estimate the Cohen’s Kappa measure of agreement for the interpretation of the change in relative risk of dengue between the global and annual time scales.

## Methods

### Cases of dengue disease from Bucaramanga, Colombia

The city of Bucaramanga, Colombia is located at coordinates $7^{\circ }7'07''$N–$73^{\circ }06'58''$ W, at 959 m above sea level. It covers an urban area of 27 km$^{2}$ and it has a population of 527,913 people in 2016, living in 220 neighborhoods nested in 17 communes. While Colombia presented an incidence rate of 436 cases per 100,000 persons in 2010, Bucaramanga reported an incidence rate of 1359.1 per 100,000 persons. We obtained data on incident cases of dengue disease (dengue and severe dengue) from the SIVIGILA (public health surveillance system) for the urban area of Bucaramanga for the period from January 2008 to December 2015.

We geocoded and allocated every case of dengue disease to one of 293 Bucaramanga census sections (geographical unit composed by approximately 20 closed city blocks) according to the cartography of the 2005 census from the national geostatistical framework of the ‘Departamento Nacional de Estadística’ (Colombia national statistics office) [18]. For geocoding purposes, we started with a database of 30,063 cases corresponding to the notified dengue cases from health institutions in Bucaramanga to the surveillance system. The cases were obtained from the database checked for duplicates reported to the surveillance system. The dengue cases data included address, sex, age and an identification code which anonymized the name and personal identification of the case to the geocoder. From this database, we selected the cases with address of residence belonging to Bucaramanga. We discarded cases without address, cases with rural address and wrong addresses. Then, an R [19] script sent batches of addresses to the web geocoding service of ArcGIS server. The returned geocoding were checked and accepted, or revised for a new geocoding cycle. At the end of the process, we succesfully geocoded to the urban area of Bucaramanga a total of 27,301 cases, which were aggregated to the spatial scale defined above, therefore our data does not relate to an identifiable natural person thus the data subject is not identifiable.

The cases aggregated by census section were aggregated along two time scales: a global scale, running for the entire study period (2008–2015); and an annual scale, resulting in eight respective datasets for each included year.

We obtained disaggregated data by census section, sex and five-years age groups from the 2005 census and calculated annual and global crude incidence rates according to these variables. We calculated expected values for dengue case counts by multiplying the global and annual crude incidence of dengue times the population by sex and age at census section level.

### Satellite images for normalized difference vegetation index

We used satellite raster images obtained from Landsat Surface Reflectance (SR) Enhanced Thematic Mapper (ETM) 7, bands 3 and 4 (60 m resolution) for the years 2008, 2009, 2010, 2011; and from Landsat SR Operational Land Imager (OLI) and Thermal Infrared Sensor (TIRS) 8, bands 4 and 5 (30 m resolution) for years 2013, 2014, and 2015 (Landsat Surface Reflectance products courtesy of the U.S. Geological Survey). We selected Landsat multispectral images based on those with the least cloud cover. Images covered the city of Bucaramanga and were taken from row 55, path 8 or path 7, with Universal Transversal Mercator (UTM) projection, zone 18 North and datum WGS-84. From Landsat SR ETM 7, we selected images from January 2, 2008; January 27, 2009; January, 14 2010; and February 2, 2011. From Landsat SR OLI-TIRS 8, we chose images from June 16, 2013; January 10, 2014; and January 4, 2015. We did not find any suitable Landsat images for the year 2012.

### Satellite images for land surface temperature

We use Moderate Resolution Imaging Spectroradiometer (MODIS) satellite raster images from the MOD11A2 version 6 product [20] to obtain mean 8-day, per-pixel land surface temperature (LST), in a 1200 km $\times$ 1200 km grid. Each pixel value in the MOD11A2 is a simple average of all the corresponding MOD11A1 LST pixels collected within that 8-day period, with a pixel size of 1000 m $\times$ 1000 m. We selected the ‘day time surface temperature’ band.

### Image processing

For the Landsat SR 7 ETM raster images, we calculated a composite NDVI raster image for the annual satellite images using band 4 [near infra red (NIR)] and band 3 (red). For the Landsat SR 8 OLI-TIRS raster images, we calculated a composite NDVI raster image for the annual satellite images using band 5 NIR and band 4 (red). We applied the formula NDVI = (NIR band − red band)/(NIR band + red band), following Yuan et al. [21]. Due to the absence of good quality images for 2012, we created a composite NDVI image for 2012 by pixel averaging of the composite NDVI images for 2011 and 2013.

To obtain an NDVI value by census section, we superimposed a mask comprised by the polygons (census sections) from the shape file of the city of Bucaramanga, onto the composite NDVI raster image, calculating the NDVI pixel mean by census section to the composite NDVI annual satellite images. We also produced a composite NDVI image at global aggregation scale (2008–2015 period) by pixel averaging by census section of all the composite NDVI images per year. For the composite NDVI image at global scale, we followed the same masking procedure to produce NDVI pixel mean by census section applied to the composite NDVI images by year.

For the MODIS LST raster images, we first reprojected the images from sinusoidal projection to UTM 18N projection, datum WGS84, and resampled to 30 m using the Modis reprojection tool (MRT) software [22]. We then created composite LST raster images per epidemiological period by pixel averaging all the reprojected and resampled images available in every epidemiological period. We generated composite LST images per year by pixel averaging all the composite LST raster images per epidemiological period. For every composite LST raster image per year, we applied a mask comprised by the polygons (census sections) from the shape file of the city of Bucaramanga, and calculated the average LST by polygon (census section).

Finally, we created a composite LST image at global aggregation scale (2008–2015 period) by pixel averaging all the composite LST images per year. We produced LST average by census section in the composite LST image at global scale, following the same masking process to obtain LST average by census section per year. Raster image processing was done using the R software version 3.3 [19] with the raster package version 2.5-8 [23].

### Statistical models

Disease mapping is the area of epidemiology that estimates the spatial pattern in disease risk over an extended geographical region in order to identify areas at high risk [24]. Besag et al. [25] developed a Bayesian hierarchical model for spatial analysis of areal data. Let $\theta _{i}$ be the log relative risk of a contagious disease with low transmission, $O_{i}$ the observed number of cases and $E_{i}$ the expected number of cases in area *i*. Assuming $O_{i}$ are independent Poisson variables with mean $E_{i}$exp$(\theta _{i})$, where exp$(\theta _{i})$ is the relative risk of the disease and the linear predictor $\theta = t + u + v$ is adopted, where *t* is a term associated with measured covariates; and *u* and *v* are surrogates for unknown observed covariates. The $u_{i}$’s represent variables with spatial structure and the $v_{i}$’s represent spatially unstructured variables. In the hierarchical framework, prior probability distributions are assigned to *u* and *v*. For *v*, Normal prior with zero mean and variance $\lambda$, and for *u*,

1 $$\begin{aligned} p(u)\propto \left\{ -\sum _{i<j}\zeta _{ij}\phi (u_{i}-u_{j}) \right\} , \quad u \in \mathcal {R} \end{aligned}$$

where $\phi$ is a function of pairwise differences among *u*’s, and $\zeta _{ij}$ are weights equal to zero for *i* non-contiguous to *j*. Besag et al. [25] consider two options for $\phi$: $\phi (z)=z^{2}/2\kappa$ or $\phi (z)=|z|/\kappa$, where $\kappa$ is an unknown constant. Choosing the first options for $\phi$, the conditional structure of *u* follows

2 $$\begin{aligned} p(u|\kappa )\propto \frac{1}{\kappa ^{n/2}}\left\{ -\frac{1}{2\kappa }\sum _{i\sim j}\zeta _{ij}(u_{i}-u_{j})^{2} \right\} \end{aligned}$$

where $i\sim j$ represent neighbor areas, and the model is referred to as the ‘Normal intrinsic autoregression.’ For the non-zero $\zeta _{ij}$ several options are available, such as 1 for zones sharing a border and 0 otherwise, or the length of the boundary between contiguous zones [10]. The model with independent normal priors for $v_{i}$ and Normal intrinsic priors for $u_{i}$ is known as the ‘convolution model’.

We fitted Poisson log normal models for relative risk, following Besag et al. [25], to the aggregated data at annual and global scale, with observation equation,

3 $$\begin{aligned} O_{i} \sim \text{ Poisson } (E_{i}e^{\theta _{i}}) \end{aligned}$$

where $O_{i}$, $E_{i}$ and exp($\theta _{i}$) are the observed count, the expected count, and the relative risk of dengue disease, respectively, in census section *i* ($i=1,\ldots ,m, m=293$). For the linear predictor, we explored the following structures:

4 $$\begin{aligned} \theta _{i}= \left\{ \begin{array}{l} \alpha + u_{i} + v_{i} \\ \alpha + u_{i} + v_{i} + \beta _{1}\text{ NDVI }_{i} \\ \alpha + u_{i} + v_{i} + \beta _{2}\text{ LST }_{i}\\ \alpha + u_{i} + v_{i} + \beta _{1}\text{ NDVI }_{i} + \beta _{2}\text{ LST }_{i}\\ \end{array} \right. \end{aligned}$$

where the $u_{i}$ are spatially correlated effects with Normal intrinsic conditional autoregressive (ICAR) priors distribution with precision parameter $\tau _{u}$, and the $v_{i}$ are the spatially uncorrelated effects with Normal prior distribution with zero mean and precision parameter $\tau _{v}$. $\beta _{j}$ ($j=1,2$) are normally distributed fixed coefficients for NDVI and LST, with zero mean and precision 100. Uniform (0,1) hyperpriors were assigned to $\tau _{u}^{-1/2}$ and $\tau _{v}^{-1/2}$.

Next, we fitted models with spatially correlated effects with Leroux [26] Normal conditional autorregressive (CAR) prior distribution and fixed coefficients for the covariates,

5 $$\begin{aligned} \theta _{i}=\left\{ \begin{array}{l} \alpha + w_{i}\\ \alpha + w_{i} + \beta _{1}\text{ NDVI }_{i} \\ \alpha + w_{i} + \beta _{2}\text{ LST }_{i} \\ \alpha + w_{i} + \beta _{1}\text{ NDVI }_{i} + \beta _{2}\text{ LST }_{i} \\ \end{array} \right. \end{aligned}$$

The $w_{i}$ are the spatially correlated effects with Leroux Normal CAR prior distributions, with precision matrix $\tau _{w}$ with Gamma(1, 0.001) hyperpriors. Finally, models were fitted with fixed and spatially varying coefficients for the covariates with Leroux CAR priors as follow:

6 $$\begin{aligned} \theta _{i}=\left\{ \begin{array}{l} \alpha + (\beta _{1} + b_{i,1})\text{ NDVI }_{i} + (\beta _{2} + b_{i,2})\text{ LST }_{i}\\ \alpha + (\beta _{1} + b_{i,1})\text{ NDVI }_{i}\\ \alpha + (\beta _{2} + b_{i,2})\text{ LST }_{i}\\ \end{array} \right. \end{aligned}$$

where the $b_{i,j}$ are spatially varying coefficients for NDVI (*j* = 1) and LST (*j* = 2). For the linear predictor including two spatially varying coefficients $b_{i,j}$, these coefficients are modelled multivariate Normal with Leroux conditional mean vector $\mu _{i,j}$ ($j=1,2$) and precision matrix $\xi _{2 \times 2}$ following Congdon (2014) [27], where $\mu _{i,j}|\mu _{k \in \partial _{i},j} = (\rho /(1-\rho + \rho d_{i}))\sum _{k \in \partial _{i}} \mu _{k,j}$ and $\xi _{2 \times 2}=(1-\rho + \rho d_{i})\Xi _{2 \times 2}$, where the precision matrix $\Xi _{2 \times 2}$ is Wishart distributed with symmetric matrix *S* and 2 degrees of freedom.

The coefficient $\rho$ establishes the degree of spatial structure of the spatial effects $\mu _{i,j}$. When $\rho$ = 1, the Leroux prior for the spatial effects implies an Normal ICAR prior, while, $\rho$ = 0, we have an independent model [28].

For the models with linear predictor including spatially varying coefficients for only one covariate, the $b_{i,1}$ or $b_{i,2}$ space-varying coefficients for NDVI or LST are modelled with Leroux conditional mean vector $\mu _{i,j}$ ($j = 1,2$) and precision $\tau _{b_{j}}$ (*j* = 1 for NDVI or *j* = 2 for LST), with Gamma(1,0.001) hyperpriors. All models included an intercept $\alpha$ with a diffuse improper prior .

From the Poisson log Normal models, we obtain the relative risk exp($\theta _{i}$) of dengue disease by census section and calculate point-wise mean estimates and 95% credible intervals (CI). Choropleth maps were produced using the logarithm of the mean relative risk ($\theta _{i}$) and the mean spatially correlated effects $u_{i}$ by census section.

Additionally, the relative risk of dengue disease by census section was discretized as *low* or *high* risk, based on the lower bound of the 95% CI, where a value of 1 or less for the lower bound of the relative risk of dengue disease by census section indicates a *low* risk, and values exceding 1 signify a *high* risk. Choropleth maps of the discretized relative risk (DRR) of dengue disease are produced at global and annual aggregation scale.

Using the DRR of dengue disease, we calculated the Cohen’s Kappa [29] coefficients for global-to-annual or annual-to-annual agreement of *low-high* risk by census section, using the following Bayesian model adapted from Lee and Wagenmakers [30]:

7 $$\begin{aligned} \kappa&= {} (\delta - \psi )/(1 - \psi )\nonumber \\ \delta&= {} \pi _{1} + \pi _{4}\nonumber \\ \psi&= {} (\pi _{1}+\pi _{2})(\pi _{1}+\pi _{3}) + (\pi _{3}+\pi _{4})(\pi _{2}+\pi _{4})\nonumber \\ \mathbf {y}&\sim {} \text{ Multinomial }([\pi _{1},\pi _{2},\pi _{3},\pi _{4}],n)\nonumber \\ \pi _{i}&\sim {} \text{ Dirichlet }(1,1,1,1) \end{aligned}$$

where $\kappa$ is the Kappa coefficient and $\mathbf {y}$ is the vector of counts in categories from the cross tabulation of *low* and *high* risk from global-to-annual or annual-to-annual agreement. Let year$_{a}$ be one the eight study years and year$_{b}$ other year not equal to year$_{a}$, the categories are as follows: $y_{1}$ = *low* risk in global scale or year$_{a}$ and *low* risk in year$_{b}$; $y_{2}$ = *low* risk in global scale or year$_{a}$ and *high* risk in year$_{b}$; $y_{3}$ = *high* risk in global scale or year$_{a}$ and *low* risk in year$_{b}$; and $y_{4}$ = *high* risk in global scale or year$_{a}$ and *low* risk in year$_{b}$.

We first calculated the global-to-annual agreement of DRR, between the pairs of DRR from model by global aggregation scale and the models for the data at annual aggregation scale. Second, we calculated the annual-to-annual agreement of DRR between all pairs of models fitted at annual aggregation scale. For the interpretation of the Kappa coefficients, we used the categories in Table 1, from Broemeling [31].

**Table 1:** Degree of agreement for Kappa

Degree of agreement	Poor	Slight	Fair	Moderate	Substantial	Perfect
Kappa	${<}0$	0–0.20	0.21–0.40	0.41–0.60	0.61–0.80	0.81–1.00

Models were fitted with Markov Chain Monte Carlo (MCMC) using the WinBUGS software version 1.4 [32]. We utilize three chains with a burn-in period of 30,000 iterations, a final run of 10,000 iterations and thinning rate of 10, deriving a final sample of 1000 iterations by chain for the inference. To evaluate convergence, we check trace and density plots as well as the Gelman, Brooks and Rubin and Geweke tests [19]. Model selection was accomplished using the deviance, the deviance information criterion (DIC) and the number of effective parameters (p$_{D}$) [33].

## Results

### Summary statistics

Table 2 presents summary statistics for the dengue case counts, the standardized morbidity rate (SMR, the observed dengue cases divided by the expected dengue cases by census section), the NDVI, the LST and, the correlations between these variables, for the data aggregated at global and annual scales.

**Table 2:** Summary statistics for counts of dengue disease, NDVI and LST, by Bucaramanga census section, for globally and annually aggregated data, 2008–2015

Statistic	Global (2008–2015)	2008	2009	2010	2011	2012	2013	2014	2015
*Dengue disease case counts*									
Total	27,301	1936	3131	6932	896	1546	4839	4956	3065
Min.	1	0	0	0	0	0	0	0	0
Max.	433	31	56	109	17	39	115	84	58
Mean	93.2	6.6	10.7	23.7	3.0	5.3	16.5	16.9	10.5
*Standardized morbidity rate*									
Min.	0.225	0.000	0.000	0.000	0.000	0.000	0.000	0.000	0.000
Max.	4.052	8.409	6.346	5.401	12.294	5.979	4.683	5.129	5.502
Mean	1.098	1.125	1.082	1.143	1.193	1.089	1.096	1.101	1.165
*NDVI*									
Min.	0.135	0.091	0.130	0.096	0.134	0.144	0.142	0.146	0.129
Max.	0.792	0.767	0.813	0.757	0.803	0.850	0.896	0.784	0.779
Mean	0.368	0.346	0.394	0.355	0.368	0.379	0.390	0.367	0.346
*LST*									
Min.	28.8	29.3	28.8	27.6	28.4	29.1	29.3	28.9	28.5
Max.	34.0	34.2	34.9	32.9	34.2	34.5	34.6	34.8	32.8
Mean	32.1	32.3	32.5	30.7	31.8	32.4	32.2	32.9	31.7
*Linear correlation*									
Dengue-NDVI	−0.071	0.062	0.170	0.108	0.080	0.061	0.198	−0.014	0.086
Dengue-LST	0.127	0.016	−0.038	0.056	−0.063	−0.039	−0.126	0.017	−0.043
NDVI-LST	−0.629	−0.533	−0.640	−0.546	−0.583	−0.629	−0.568	−0.543	−0.522

For the global scale, a total of 27,301 cases (range by census section: 1–433) were reported and geocoded. The mean SMR for all census section was 1.098, with a minimum of 0.225 and a maximum of 4.05. The mean value of NDVI was 0.368, with a minimum of 0.135 and a maximum of 0.792. Mean LST for the aggregated data was $32.1\,^\circ$C, with a minimum of $28.8\,^\circ$C and a maximum of $34\,^\circ$C. Linear correlations between counts of cases of Dengue and NDVI (r = −0.071) and LST (r = 0.127) was weak, while, correlation between the NDVI and LST was moderate and negative (r = −0.629).

For the summary statistics at annual scale, 2010 was the year with the highest number of cases (n = 6932) followed by 2014 (n = 4956) and 2013 (n = 4839), with 2011 showing the lowest number of cases (896). The maximum number of cases in a census section ocurred in 2013 (n = 115), followed by 2010 (109) and 2014 (84). For the annual SMR, the year 2011 presented the highest average SMR (1.193) followed by 2010 (SMR = 1.143) and 2015 (SMR = 1.165).

The lowest maximum NDVI by census section corresponded to the year 2010 (NDVI = 0.757) followed, in order, by 2008 (NDVI = 0.767) and 2014 (NDVI = 0.784), while the lowest mean NDVI were for years 2008 and 2015 (mean NDVI = 0.346) followed by 2010 (mean NDVI = 0.355).

With respect to the LST, the year 2010 displayed the lowest mean temperature ($30.7\,^\circ$C), followed by 2015 ($31.7\,^\circ$C) and 2011 ($31.8\,^\circ$C), while for the rest of the years, the LST mean was close to $32\,^\circ$C.

The linear correlations between dengue case counts and NDVI or LST were low, whereas the most pronounced correlation was for 2013: between dengue and NDVI, r = 0.198; and between dengue and LST, r = −0.126. At an annual scale, the correlation between NDVI and LST was moderate and inverse for all years, with the strongest correlations for 2009 (r = −0.640) and 2012 (r = −0.629).

### Model selection

Table 3 shows the selection statistics deviance, number of effective parameters (p$_{D}$) and DIC, for the models fitted at global and annual aggregation scales in Bucaramanga.

**Table 3:** Information criterion statistics, for relative risk models of dengue disease, 2008–2015

Model	Deviance	p$_{D}$	DIC	Deviance	p$_{D}$	DIC	Deviance	p$_{D}$	DIC
	Global scale 2008–2015			2008			2008		
$u_{i} + v_{i}$	2098.0	268.3	2366.3	1290.5	168.5	1459.0	1428.0	191.9	1619.9
$u_{i} + v_{i} + \beta _{1}$	*2097.2*	*267.4*	*2364.6*	1291.3	168.7	1460.0	*1427.7*	*190.7*	*1618.4*
$u_{i} + v_{i} +\beta _{2}$	2097.3	267.4	2364.7	1289.8	168.2	1458.0	1427.0	192.2	1619.2
$u_{i} + v_{i} + \beta _{1} + \beta _{2}$	2097.8	267.9	2365.8	1289.5	168.7	1458.2	1427.9	191.7	1619.6
$w_{i}$	2098.1	268.9	2367.1	1296.2	163.3	1459.5	1428.8	196.0	1624.8
$w_{i} + \beta _{1} + \beta _{2}$	2098.1	268.6	2366.7	1295.7	163.5	1459.1	1429.0	194.5	1623.5
$w_{i} + \beta _{1}$	2098.3	268.9	2367.2	*1294.5*	*163.3*	*1457.8*	1429.1	194.5	1623.6
$w_{i} + \beta _{2}$	2098.0	269.0	2367.0	1296.2	163.7	1459.9	1428.7	196.0	1624.6
$\beta _{1} + b_{i,1} + \beta _{2} + b_{i,2}$	2108.4	261.8	2370.2	1323.6	157.2	1480.8	1443.5	186.9	1630.5
$\beta _{1} + b_{i,1}$	2113.8	257.0	2370.8	1346.2	140.6	1486.9	1465.5	175.7	1641.3
$\beta _{2} + b_{i,2}$	2279.2	267.1	2546.3	1423.8	133.7	1557.5	1616.1	158.7	1774.8
	2010			2011			2012		
$u_{i} + v_{i}$	1684.3	233.5	1917.7	1063.3	120.5	1183.8	1198.4	164.8	1363.2
$u_{i} + v_{i} + \beta _{1}$	*1683.9*	*232.6*	*1916.5*	1063.5	120.6	1184.1	1199.6	165.6	1365.2
$u_{i} + v_{i} +\beta _{2}$	1684.4	233.8	1918.2	1063.0	119.9	1182.9	1198.2	165.6	1363.8
$u_{i} + v_{i} + \beta _{1} + \beta _{2}$	1683.7	232.8	1916.5	1063.6	121.4	1185.0	1198.5	165.9	1364.5
$w_{i}$	1685.6	236.9	1922.5	1072.2	115.1	1187.3	1208.2	163.3	1371.4
$w_{i} + \beta _{1} + \beta _{2}$	1686.0	236.0	1922.0	1071.0	117.4	1188.4	1207.7	163.6	1371.3
$w_{i} + \beta _{1}$	1686.0	236.5	1922.6	1072.2	115.9	1188.2	1208.4	163.3	1371.7
$w_{i} + \beta _{2}$	1684.0	236.2	1920.2	1071.8	115.9	1187.7	1207.8	163.8	1371.7
$\beta _{1} + b_{i,1} + \beta _{2} + b_{i,2}$	1694.3	227.6	1921.8	1065.9	121.0	1186.9	1206.8	156.6	1363.4
$\beta _{1} + b_{i,1}$	1699.1	220.5	1919.6	*1077.6*	*100.7*	*1178.4*	*1214.9*	*146.8*	*1361.8*
$\beta _{2} + b_{i,2}$	1823.0	228.0	2051.0	1168.5	69.3	1237.8	1332.8	119.6	1452.4
	2013			2014			2015		
$u_{i} + v_{i}$	1556.9	216.5	1773.4	1582.5	216.7	1799.2	1443.9	193.5	1637.4
$u_{i} + v_{i} + \beta _{1}$	1557.8	215.3	1773.1	*1581.9*	*216.4*	*1798.3*	1443.2	193.3	1636.5
$u_{i} + v_{i} +\beta _{2}$	1557.4	216.2	1773.6	1582.5	216.6	1799.2	1443.4	193.4	1636.8
$u_{i} + v_{i} + \beta _{1} + \beta _{2}$	1557.7	215.4	1773.1	1581.8	217.3	1799.1	1443.4	193.8	1637.2
$w_{i}$	1560.0	216.5	1776.5	1586.4	220.0	1806.4	1447.1	197.5	1644.6
$w_{i} + \beta _{1} + \beta _{2}$	1559.9	215.4	1775.3	1584.6	219.3	1803.9	1447.1	197.2	1644.3
$w_{i} + \beta _{1}$	1560.0	215.0	1775.0	1585.2	219.4	1804.7	1446.8	196.9	1643.7
$w_{i} + \beta _{2}$	1560.4	216.8	1777.2	1584.8	219.4	1804.2	1447.1	197.2	1644.3
$\beta _{1} + b_{i,1} + \beta _{2} + b_{i,2}$	1570.4	202.8	1773.1	1589.6	209.7	1799.3	1452.5	179.6	1632.1
$\beta _{1} + b_{i,1}$	*1579.3*	*192.9*	*1772.2*	1597.2	202.3	1799.5	*1452.9*	*176.2*	*1629.1*
$\beta _{2} + b_{i,2}$	1912.6	178.8	2091.4	1764.9	200.9	1965.8	1635.3	160.2	1795.5

*p* $_{D}$ is defined as number of effective parameters

In general, the convolution models with CAR priors fitted the data better than the models with Leroux CAR priors, evidenced by the smallest deviance in the convolution models. For the data at global aggregation scale, the model with spatially correlated and uncorrelated effects with fixed coefficient for NDVI ($u_{i} + v_{i} + \beta _{1}$) presented the smallest DIC (DIC = 2364.6). For the year 2008, the smallest DIC was for the model with spatial effects with Leroux CAR priors and fixed coefficient for NDVI ($\beta _{1} + w_{i}$) (DIC = 1457.8). For the years 2009, 2010, and 2014, we selected the models with correlated and uncorrelated spatial effects plus a fixed coefficient for NDVI ($u_{i} + v_{i} + \beta _{1}$), which presented the smallest DIC values of 1618.4, 1916.5, and 1798.3, respectively. For the years 2011, 2012, 2013, and 2015, the selected models contained space-varying coefficients for NDVI with Leroux CAR priors and a fixed coefficient for NDVI ($\beta _{1} + b_{i,1}$), displaying the smallest DIC values of 1178.4, 1361.8, 1772.2, and 1629.1 respectively.

### Parameter estimates of the selected model at global aggregation scale, 2008–2015

**Fig. 1:**
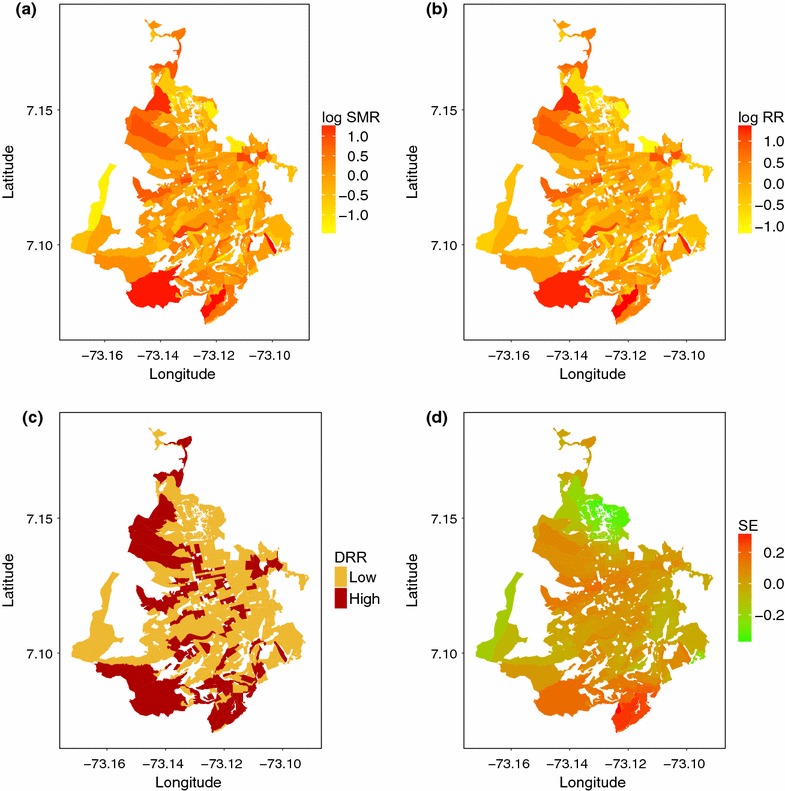
**a** Logarithm of the standardized morbidity rate (log SMR); **b** logarithm of the mean relative risk (log RR) of dengue disease; **c** discretized relative risk (DRR$_{i}$); and **d** mean spatial effects (SE) $u_{i}$, by census section for the data aggregated at global scale for the period 2008–2015

The selected model at global scale was the convolution model with fixed coefficient for NDVI ($u_{i} + v_{i} + \beta _{1}$). Figure 1 shows the map of the SMR logarithm, the map of the logarithm of the mean relative risk of dengue disease $\theta _{i}$, the DRR of dengue disease, and the spatially correlated effects ($u_{i}$) from the model at global scale. The log mean relative risk of dengue disease shows high risk clusters in the south and north-west census sections of the city. The DRR map presents the areas where the 95% CIs for the relative risk do not include 1, and the map of spatial effects displays spatial correlation at the south of the city.

### Parameter estimates from models at annual aggregation scale, 2008–2015

Table 4 presents the mean point-wise parameters estimates and 95% CI from the selected models fitted at the annual aggregation scale.

**Table 4:** Parameter estimates (point-wise mean and 95% CI) from the selected model at annual scale, 2008–2015 period

Parameter	Year			
	2008	2009	2010	2011
$\alpha$	−0.173 (−0.399, 0.059)	−0.305 (−0.494, −0.115)	−0.19 (−0.363, −0.007)	0.108 (−0.12, 0.332)
$\sigma _{u}$	0.811 (0.658, 1.008)	0.323 (0.153, 0.512)	0.342 (0.199, 0.494)	
$\sigma _{v}$		0.457 (0.372, 0.543)	0.45 (0.386, 0.518)	
$\beta _{1}$	0.294 (−0.214, 0.814)	0.589 (0.168, 1.024)	0.43 (−0.041, 0.885)	−0.398 (−1.042, 0.249)
$\sigma _{b_{i,1}}$				1.399 (1.063, 1.835)
$\rho$	0.486 (0.217, 0.868)			0.062 (0.002, 0.236)

Parameter	2012	2013	2014	2015
$\alpha$	0.116 (−0.11, 0.346)	−0.08 (−0.224, 0.071)	−0.093 (−0.28, 0.083)	0.049 (−0.138, 0.223)
$\sigma _{u}$			0.5 (0.346, 0.684)	
$\sigma _{v}$			0.404 (0.322, 0.483)	
$\beta _{1}$	−0.635 (−1.367, 0.12)	0.002 (−0.496, 0.502)	0.058 (−0.39, 0.536)	−0.222 (−0.867, 0.456)
$\sigma _{b_{i,1}}$	2.068 (1.601, 2. 711)	1.785 (1.469, 2.188)		2.034 (1.666, 2.506)
$\rho$	0.279 (0.081, 0.592)	0.314 (0.146, 0.567)		0.316 (0.141, 0.575)

The selected model for 2008 was the model with spatially correlated effects with Leroux CAR priors plus fixed coefficient for NDVI ($w_{i} + \beta _{1}$). The point-wise mean estimate for the NDVI fixed coefficient is positive (0.294), and the 95% CI include zero (−0.214, 0.814), suggesting a weak association between dengue and NDVI by census section, at the same time, the point mean estimate of $\rho$ is 0.486, which denotes moderate spatially correlated effects $w_{i}$ in the relative risk of dengue disease.

**Fig. 2:**
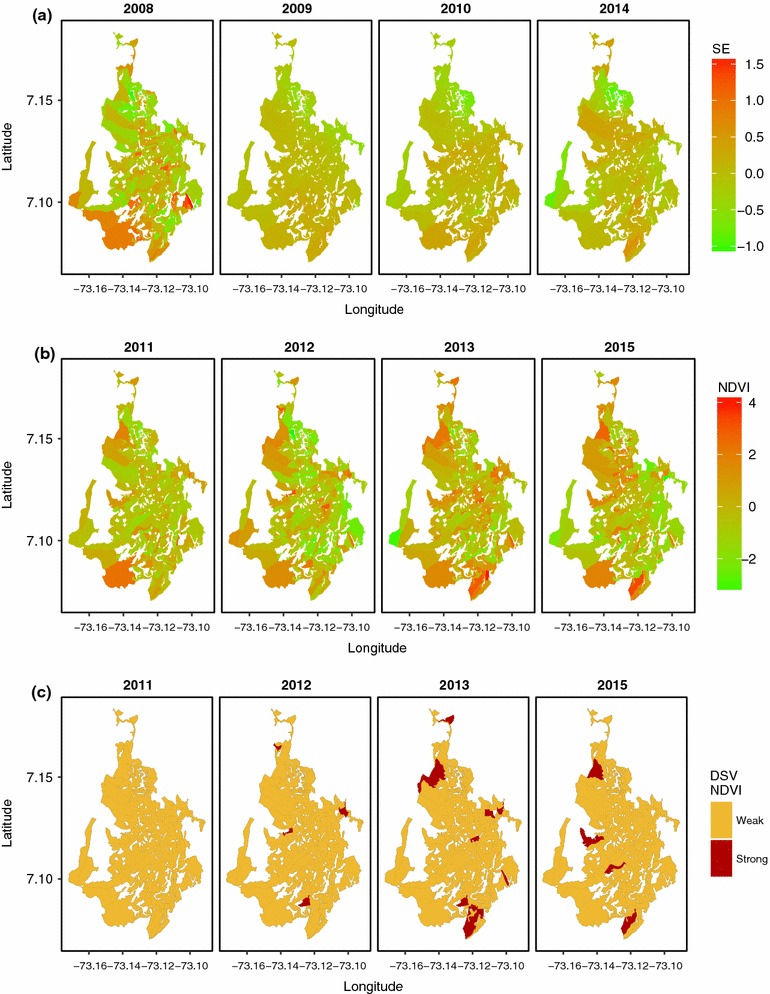
**a** Mean spatial effects (SE) $w_{i}$ (2008) and $u_{i}$ (2009, 2010, and 2014) from the selected models at annual aggregation scale; **b** mean space-varying NDVI coefficients ($\beta _{1}+b_{i,1}$); and **c** discretized space-varying (DSV) NDVI coefficients ($\beta _{1}+b_{i,1}$) for years 2011, 2012, 2013, 2015, from models at annual aggregation scale

The convolution model plus fixed coefficient for NDVI was the selected model for the years 2009, 2010, and 2014 ($u_{i} + v_{i} + \beta _{1}$). The point-wise mean and 95% CI estimates of the fixed coefficients for NDVI for years 2009, 2010, and 2014 show a strong, positive association between Dengue and NDVI for 2009 (0.589; 95% C.I: 0.168, 1.024), but not for 2010 and 2014. The mean of the spatially correlated effects $w_{i}$ (2008) and $u_{i}$ (2009, 2010 and 2014) are presented in Fig. 2a. We observe the highest values of the mean spatial effects for the south of the city in 2008, but a scattered pattern of the mean of the spatially correlated effects for the rest of the years.

The model with fixed coefficient and space-varying coefficients for NDVI was selected for years 2011, 2012, 2013, and 2014 ($\beta _{1} + b_{i,1}$). The fixed coefficients for NDVI have to be accompanied by the space-varying coefficients, to be fully interpreted. The mean estimates for the fixed coefficient plus the space-varying coefficients for NDVI ($\beta _{1} + b_{i,1}$) are presented in the Fig. 2b. We discretized the space-varying coefficients in a similar way as the discretized relative risk (DRR). We considered the association between the NDVI and dengue by census section to be *weak* when the lower bound of the 95% CI was 1 or less and, to be *strong* otherwise (Fig. 2c). The discretized space-varying (DSV) NDVI coefficients enabled us to identify census sections where there was strong association between dengue incidence and NDVI. For 2012, 2013, and 2015, we observe a strong asssociation in 4 to 8 census sections, while for 2011, the discretized effect was so low that there were no census sections showing an association between the covariate and dengue disease.

### Mapping of relative risk of dengue disease, from models by annual scale

In this section, we begin by presenting the maps for the logarithm of the mean relative risk of dengue disease, and then we display the maps of the DRR of dengue, from the models selected by year 2008–2015.

**Fig. 3:**
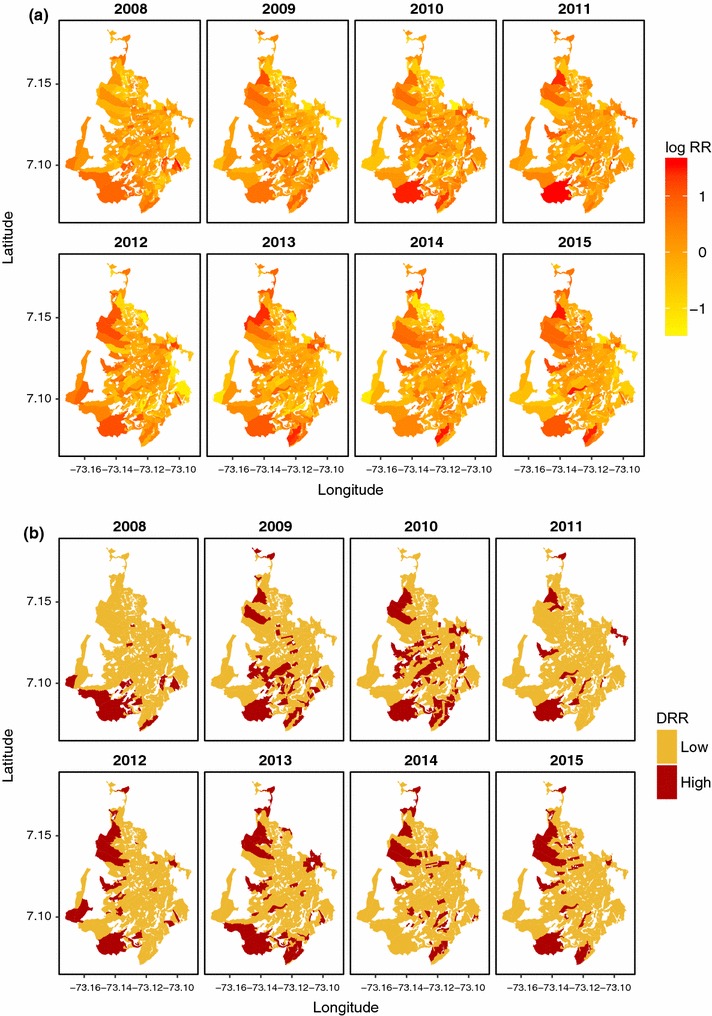
**a** Logarithm of the mean relative risk (log RR) of dengue disease , from models at annual aggregation scale 2008–2015; **b** discretized relative risk (DRR) of dengue disease, 2008–2015

Figure 3a presents the logarithm of the mean relative risk (Log RR) by year, for the period 2008–2015. The smoothed estimates of the Log RR let us discern patterns of the disease distribution in Bucaramanga. We observe similar patterns for 2009, 2010, and 2011, with clusters of high relative risk in the southern and northwestern census sections. For 2008, 2012, 2013 and 2015, we observe slightly fewer zones with high relative risk. Estimates of relative risk of dengue for 2014 presented a great number of high-risk census sections in the northwestern, southern and central zones of the city.

The maps of DRR of dengue disease help us to identify rapidly those census sections presenting the highest risk for each year (Fig. 3b). The areas along the southern and northwestern edges of the city show a consistent tendency towards high relative risk, while the center generally presents a low risk, with some exceptions.

### Kappa coefficient for the global-toa-annual and annual-to-annual agreement of DRR of dengue disease

We present estimates for the Kappa coefficient (point-wise mean and 95% CI) for global-to-annual and annual-to-annual agreement of DRR of dengue disease in Table 5. We interpreted the Kappa coefficient for agreement based on the coverage of the 95% CI over the categories of ‘degree of agreement’ from Table 1. Firstly, we established the global-to-annual agreement of DRRs of dengue disease. Secondly, we determined the annual-to-annual agreement of the DRR of dengue disease.

From Table 5, we interpreted the global-to-annual agreement in DRR at years 2008, 2009, 2012, and 2015 to be *poor to fair*, while the agreement was *poor to slight* for 2011. Agreeement of DRR of dengue disease was *slight to moderate* between the model at global scale and models for 2013 and 2014, and, *substantial to moderate* for the global scale model and the model for 2010.

For the annual-to-annual agreement of DRR, from 2009 to 2010, there was *poor to fair* agreeement of DRR. From years 2011 to 2012 and 2012 to 2013, the agreement of DRR for dengue disease was *slight to moderate*. Additionally, the agreement was *slight to fair* from 2010 to 2011, 2013 to 2014, and 2014 to 2015. Finally, annual-to-annual agreement of DRR between other year pairs was almost always *poor to slight*.

**Table 5:** Kappa coefficient (point-wise mean and 95% CI) for global-to-annual and annual-to-annual agreement of discretized relative risk (DRR) of dengue by census sections, from models at global and annual aggregation scales in Bucaramanga, 2008–2015

Year	Global scale	Annual scale						
		2009	2010	2011	2012	2013	2014	2015
2008	0.231	0.146	0.107	0.087	0.142	0.115	0.003	0.042
	(0.124, 0.347)	(0.021, 0.288)	(0, 0.235)	(−0.027, 0.251)	(0.007, 0.303)	(−0.003, 0.255)	(−0.087, 0.126)	(−0.059, 0.173)
2009	0.327		0.271	0.207	0.207	0.226	0.141	0.097
	(0.207, 0.448)		(0.144, 0.402)	(0.078, 0.36)	(0.072, 0.355)	(0.094, 0.366)	(0.012, 0.281)	(−0.02, 0.234)
2010	0.513			0.143	0.158	0.294	0.134	0.075
	(0.397, 0.621)			(0.043, 0.261)	(0.046, 0.284)	(0.162, 0.425)	(0.013, 0.264)	(−0.035, 0.202)
2011	0.166				0.293	0.279	0.185	0.196
	(0.079, 0.271)				(0.116, 0.486)	(0.14, 0.431)	(0.054, 0.341)	(0.062, 0.357)
2012	0.254					0.363	0.225	0.269
	(0.144, 0.369)					(0.212, 0.513)	(0.085, 0.378)	(0.121, 0.424)
2013	0.466						0.219	0.231
	(0.352, 0.577)						(0.089, 0.361)	(0.1, 0.375)
2014	0.307							0.25
	(0.188, 0.428)							(0.112, 0.393)
2015	0.22							
	(0.099, 0.34)							

## Discussion

In this study we applied Bayesian hierarchical models for spatial analysis of areal data to the estimation of relative risk of dengue disease in a Colombian city. We chose this particular city for its high incidence of dengue disease, in 2008–2015. We fitted models at global and annual scales for the study period. The hierarchical models included covariates (NDVI and LST) obtained from satellite raster images.

From the descriptive statistics, we found low correlation between the covariates and the dengue case counts by census section. NDVI and LST were moderately correlated by census sections at global and annual scales. Two main models were fitted: first the convolution model (spatially correlated effects with Normal ICAR priors, and uncorrelated spatial effects with Normal zero mean priors and precision $\tau _{v}$); and second, spatially correlated effects model with Leroux Normal CAR priors. We used those structures with or without covariates. The covariates effects were modeled as fixed coefficients with Normal prior distributions with zero mean and high precision, or space-varying coefficients with Leroux Normal CAR priors.

We used MCMC to fit the models, and selected the models for inference, applying DIC measures. All the selected models (at global and annual scale) included NDVI fixed coefficients (2008, 2009, 2010, and 2015), and NDVI space-varying coefficients (2011, 2012, 2013, and 2014).

The convolution model was selected at global and annual scale for 2009, 2010, and 2014. The models with spatially correlated effects with Leroux Normal CAR priors were selected for 2008, 2011, 2012, 2013, and 2015. We illustrated the relative risk of dengue disease using two types of maps. First, we produced maps of the logarithm of the mean relative risk, containing smoothed estimates of relative risk, allowing us to distinguish clusters of high relative risk at global and annual scales. Second, we created maps of DRR of dengue disease, allowing us to identify zones where the risk is higher than in zones with 95% CIs including 1.

We employed satellite images from two sources (Landsat and MODIS) to calculate NDVI and LST data at areal level (census section). We were interested in establishing association between the information from raster images and dengue incidence at global and annual scale according to census section. The selected models for inferences did include NDVI but not LST. Parameter estimates (point-wise mean and 95% CI) for the association of the NDVI and dengue disease are mainly positive; however, they only reveal a strong association between dengue and NDVI, in 2009 (95% CI not including zero). Our results were different from some results in the literature. In Costa Rica, Troyo et al. [34] found negative coefficients for NDVI suggesting an inverse association with relative risk of dengue disease. They reported NDVI from satellite images at different resolutions, finding an association between high NDVI and low incidence of dengue. Meza-Ballesta et al. [35] reported the association between dengue incidence and high air temperature, high rainfall, and vegetation deterioration. Araujo et al. [36] analyzed dengue incidence in São Paulo, Brazil. They associated thermal remote sensing images, census data, and dengue incidence, and their findings point to low dengue incidence in areas with high vegetation cover, and high incidence in areas with low vegetation coverage and land surface temperatures above $29\,^{\circ }\mathrm {C}$; however Nazri et al. [37] did not find NDVI to be a major factor influencing dengue incidence in Malaysia. Qi et al. [38] found that the associations between cases of Dengue disease and population density, GDP per capita, road density, and NDVI were nonlinear, and the risk of dengue disease declined gradually with rising NDVI.

These reports involving NDVI as a covariate did not use areal data, or the aggregation resolution was higher than the resolution used in our study. Additionally, at high spatial scale, the link between NDVI and rainfall variability in zones with epidemiological reports of dengue or malaria has been reported across South America [39], contributing to a better understanding of climate, environment, and epidemics facilitating the implementation of local and regional health early warning systems. In this context, our study contributes to understand the relationship between NDVI and incidence of dengue disease at a finer resolution.

As limitations of the satellite data employed in our study, we noted that for the period 2008–2011, we had to impute the NDVI in some census sections because of known issues with the sensor from Landsat ETM 7. We employed a convolution model to make the imputation of the missing NDVI values. For the NDVI data, the pixel resolution was 30 m, while the pixel resolution of the MODIS LST data was 1000 m, resampled to 30 meters, which makes some census section highly associated with respect to the LST. Additionally, for LST, we used single raster image per year, which is a composite of around 48 MODIS images. Moreover, finding high-quality Landsat images for the period was difficult, mainly because of cloud cover. In addition, we used a non-conventional method to treat information from satellite images, as compared to the literature, because we employed continuous variables such as NDVI and LST in space obtaining areal inputs as covariates, using the mean value for every census section.

With respect to the data quality on dengue, we employed official data, aggregating the cases into census sections acknowledging factors such as age or sex by means of internal standardization. We consider our dengue case counts to be high-quality data given that the surveillance system is based on compulsory reporting physicians in Colombia at all service levels, but keeping in mind critic considerations regarding possible underreporting as shown by Romero-Vega et al. [40].

For the spatial aggregation level and census data, we based our population estimates on the 2005 census, with the assumption that city’s population has been fairly stable throughout the study period. We recognize the possible bias produced by the use of 2005 data for estimation of the expected values for the study period. This is one of the challenges of the study, but it is not only for the disease under study. At the time of the realization of this study, there were not updated data for population living in Bucaramanga, at census section level. The Colombian government updates its census every ten years. It was programmed a census for 2016, but was not developed. We consider this finding extremely important, if public health authorities are interested to provide information, not only for temporal analysis (as is currently done) but also for spatial analysis at finer resolution scales (census block, section or sector). This study provides the space to recommend to the Colombian authorities, to build real-time registries of population, which support evaluation, decision making and intervention, not only for dengue disease, but for all the notifiable diseases. Additionally, we are aware that the spatial aggregation scale changes the conclusion from areal data as shown by Khormi and Kumar [41], as does the choice of neighborhood structure [10]. The spatial aggregation scale used in this study corresponds to the spatial scale supplied by the official cartography from the 2005 census.

Together with the generation of relative risk maps and the evaluation of satellite data associated with incidence of dengue disease, we determined whether there was an association in the DRR of dengue disease by census section from year to year, and from the risk at global scale and annual scale. To this end, we employed the Kappa coefficients to define the global-to-annual agreement of risk when the relative risk is discretized as *low* or *high* risk, estimating the Kappa coefficients, using a Bayesian model. We have found substantial to moderate agreeement between the DRR at global scale and the year 2010, and moderate to fair agreeement for 2013 and 2014. For the rest of the global-to-annual agreement or all the annual-to-annual agreement of the DRR for consecutive years, we found slight to fair agreement, reflecting a volatile pattern of dengue risk by census section from year to year. The main results for the annual-to-annual agreement of the discretized relative risk for non consecutive year, point to low agreeement between census sections in terms of risk level between years.

## Conclusions

We applied disease mapping models at small spatial scale in a Colombian city with fixed and space-varying coefficients for covariates derived of satellite images. We found the NDVI associated to high dengue risk by census section. The modeling process produced relative risk maps of dengue disease, allowing us to identify areas with high risk in the city. We compared the concordance of high risk by census section between the global aggregated model and the annual aggregated models, and between years in the 2008–2015 period. We found in general, slight to fair agreement in high risk. The information obtained by the use of disease mapping models is not currently available to public health authorities in Colombia. We highlight the importance of transforming raw spatial data into relative risk maps of dengue disease for planning and implementation of public health strategies. The map quality depends on high-quality population data at the selected spatial aggregation scale, which are sometimes difficult to obtain; and the geocoding process to allocate every case to a correct spatial coordinate. Information from satellite images improves the output of the spatial modeling, by associating environmental variables to the dengue incidence. For future research we are interested to apply disease mapping models in similar datasets from municipalities in Colombia. The main limitation is the cost of geocoding the data from the official records in terms of work-time and accurate geocoding. However, after the geocoding task is finished, the data will be available not only to spatial analysis, but also to temporal and spatiotemporal analysis, including the covariates with constant or time-varying coefficients[42]. We would like to present the method to the public health authorities from diverse surveillance offices in Colombia, and test the field applicability of the disease mapping models discussed in our study. Finally, we also are interested to apply disease mapping methods based on hierarchical Bayesian models to map the relative risk not only for dengue disease, but also for arboviral diseases like Chykungunya and Zika at small spatial scale in Colombia, to support public health authorities in disease surveillance and control strategies.

## Abbreviations

CAR: conditional autorregressive
CI: credible interval
DANE: Departamento Nacional de Estadística
DIC: deviance information criterion
DRR: discretized relative risk
DSV: discretized space-varying
ICAR: intrinsic conditional autoregressive
GIS: geographic information system
LST: land surface temperature
MODIS: moderate resolution imaging spectroradiometer
NDVI: normalized difference vegetation index
SIVIGILA: Sistema de Vigilancia en Salud Pública
SMR: standardized morbidity rate
